# The *IGH* locus relocalizes to a “recombination compartment” in the perinucleolar region of differentiating B-lymphocytes

**DOI:** 10.18632/oncotarget.16941

**Published:** 2017-04-07

**Authors:** Andrey Pichugin, Olga V. Iarovaia, Alexey Gavrilov, Ilya Sklyar, Natalja Barinova, Aleksandr Barinov, Evgeny Ivashkin, Gersende Caron, Said Aoufouchi, Sergey V. Razin, Thierry Fest, Marc Lipinski, Yegor S Vassetzky

**Affiliations:** ^1^ UMR8126, CNRS, Université Paris Sud Paris Saclay, Institut Gustave Roussy, Villejuif, France; ^2^ Institute of Gene Biology, Russian Academy of Sciences, Moscow, Russia; ^3^ LIA 1066, Laboratoire Franco-Russe de Recherche en Oncologie, Villejuif, France; ^4^ Department of Experimental Neurocytology, Research Center of Neurology, Branch of Brain Research, Moscow, Russia; ^5^ INSERM U1236, CHU de Rennes, Université Rennes 1, Rennes, France; ^6^ UMR8200 CNRS, Université Paris-Sud, Institut de Cancérologie Gustave Roussy, Villejuif, France; ^7^ Moscow State University, Moscow, Russia; ^8^ Peter the Great St. Petersburg Polytechnic University, St. Petersburg, Russia

**Keywords:** immunoglobulin genes, chromatin, recombination, differentiation

## Abstract

The immunoglobulin heavy chain (*IGH*) gene loci are subject to specific recombination events during B-cell differentiation including somatic hypermutation and class switch recombination which mark the end of immunoglobulin gene maturation in germinal centers of secondary lymph nodes. These two events rely on the activity of activation-induced cytidine deaminase (AID) which requires DNA double strand breaks be created, a potential danger to the cell. Applying 3D-fluorescence in situ hybridization coupled with immunofluorescence staining to a previously described experimental system recapitulating normal B-cell differentiation *ex vivo*, we have kinetically analyzed the radial positioning of the two *IGH* gene loci as well as their proximity with the nucleolus, heterochromatin and γH2AX foci. Our observations are consistent with the proposal that these *IGH* gene rearrangements take place in a specific perinucleolar “recombination compartment” where AID could be sequestered thus limiting the extent of its potentially deleterious off-target effects.

## INTRODUCTION

For a functional immunoglobulin to be synthesized in a mature B lymphocyte, one of its two immunoglobulin heavy chain (*IGH*) genes must first be rearranged during the so-called V(D)J recombination process which takes place in bone-marrow B-cells. This B-cell then circulates through the blood to a secondary lymphoid organs where the rearranged *IGH* gene locus is further modified. Fine tuning to the immunogen relies on somatic hypermutation (SHM) events occurring within the variable part of the *IGH* gene (reviewed in [1]). This is followed by a last intragenic recombination called class-switch recombination (CSR) which determines the final isotype of the antibody eventually produced in the mature plasma cell [2]. At each of these three steps of *IGH* gene rearrangement, DNA double strand breaks (DSBs) are created through enzymatic activities performed by the RAG1/2 complex in bone marrow, and the result of activation-induced cytidine deaminase (AID) in lymph nodes [3]. Because genomic DSBs are particularly dangerous to the cell, the off-target activity of these enzymes must be minimized in order to prevent deleterious genomic rearrangements and cell apoptosis [4–6]. One way to limit off-target activities is to strictly restrict the localization of the corresponding enzymes to a specific compartment (reviewed in [7]). Among others, nucleoli, nuclear speckles [8], PML [9] and Cajal bodies [10], histone loci [10], Polycomb [11], insulator bodies [12] as well as transcription [13], replication [14] and repair [15] factories have been identified and characterized as compartments where dedicated factors accumulate to carry various DNA transactions (reviewed in [16, 17]. It can then be assumed that the positioning of genes relative to such compartments will affect their functional state and activity. In accordance, changes in gene activity are expected to occur concomitantly with a relocalization to specific nuclear compartments.

In mice, the nuclear compartmentalization of the productive and non-productive *IGH* alleles changes during B-cell maturation. The non-productive *IGH* allele remains close to the nuclear periphery while its productive counterpart occupies a more central position [18–20]. This may be linked to the regulation of *IGH* allelic expression and recombination [18–20]. However, the nuclear positioning of the *IGH* locus has been less studied in humans. Since among mammals, the organization of chromatin tends to be species-specific - for instance, heterochromatin clusters are prominent in mice but not in humans - we have addressed the nuclear localization of the two *IGH* alleles in human B-cells undergoing maturation. To do so, we have made use of a system developed by Fest and collaborators whereby the maturation of B-cells is recapitulated *in vitro* [21]. In this system, human B-cells isolated from peripheral blood at Day_0_ are activated and induced to proliferate in the presence of cytokines and monoclonal antibodies. Differentiating cells are harvested starting at Day_4_ (see the Material and Methods). With this methodological approach, we have explored the hypothesis that both SHM and CSR take place in a specific nuclear compartment containing AID and all recombinogenic agents necessary. This hypothesis was based on the observation that RAG1/2 and AID are stored in the nucleolus when overexpressed [22, 23]. In addition, we have recently found that the human *IGH* locus localizes to the perinucleolar region in both B-cell lymphomas [24] and nuclei of activated B-cells [22]. Applying 3D-fluorescence *in situ* hybridization (3D-FISH) to the Fest system, the *IGH* locus visualized at successive stages of B-cell maturation was indeed found to associate with the nucleolus concomitantly with SHM and CSR, a time when the *IGH* gene locus underwent DNA damage while interacting with perinucleolar AID.

## RESULTS

### Radial positioning and chromatin environment of the *IGH* locus during maturation of human B-cells

**Figure 1:**
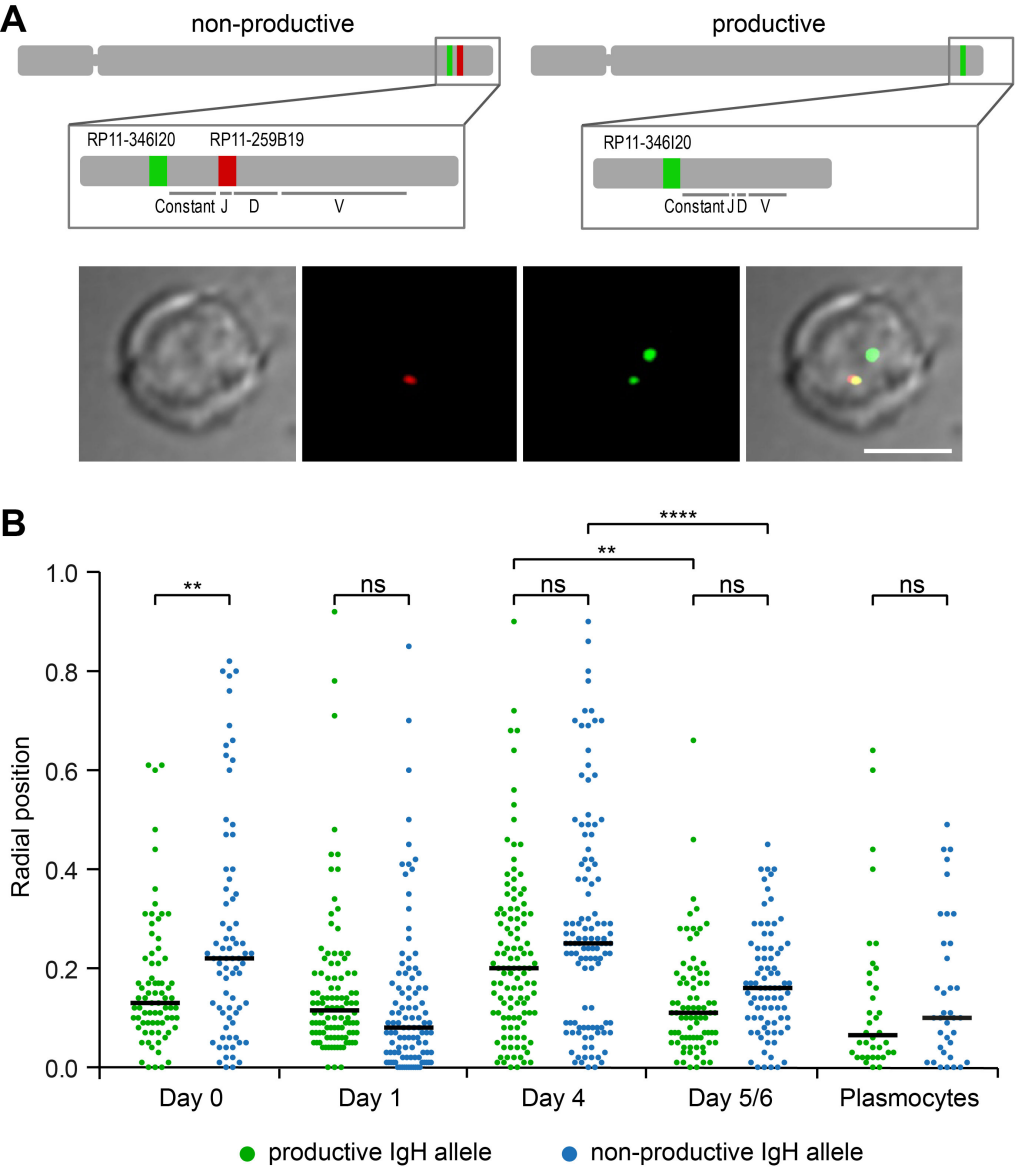
Radial positions of productive and non-productive *IGH* alleles in human B-lymphocytes **A**. A productive *IGH* allele can be discriminated by FISH in B-cells after V(D)J recombination. Top, a schematic representation of human chromosome 14 and two different *IGH* loci: an *IGH* allele with a germline structure of *IgH* locus contains two genomic fragments stained by BACs RP11-346I20 (green) and RP11-259B19 (red); the *IGH* allele which underwent V(D)J recombination and expresses functional antibodies hybridizes only with RP11-346I20 (green) which is localized upstream from constant segment. Bottom, a typical image of FISH and immunostaining is shown. Scale bar = 5 μm. **B**. Radial positions of productive and non-productive *IGH*-alleles measured in the nuclei of Day_0_ cells (n = 75), Day_1_ cells (*n* = 102), Day_4_ cells (*n* = 113), Day_5/6_ cells (*n* = 81), and Plasmocytes (*n* = 36). Black line shows median, 0 - nuclear center, 1 - nuclear periphery. *P*-values were calculated using Tukey's multiple comparison test (ns, non-significant; ** <0.01, ****<0.0001); adjusted p-values for all pairs of means are presented in Table S1. Distances were measured as described in Materials and methods. Scatter plots represent the distribution of FISH signals in the nuclear space. The nuclei were divided into concentric spheres with the equal volume.

The radial distribution of the human *IGH* gene loci was studied at the various steps of B-cell differentiation recapitulated *in vitro* in the Fest system. 3D-FISH images were produced and computer analyzed as previously described [24]. In order to distinguish between the productive and non-productive alleles, two probes were used, one detecting the constant region (green), the other a proximal part of the J region (red) which is deleted during V(D)J recombination. Thus, the productive allele is revealed as a green spot while the non-recombined allele appears either yellow if the two probes are totally superimposed, or with the red and green signals partially overlapping or separate but still in close proximity to each other (Figure 1A). It can be seen in Figure 1B that the radial positions of the rearranged productive and non-rearranged non-productive alleles did not differ significantly except at Day_0_ in naïve B-cells. Contrasting with murine B-cells where the non-productive allele has been reported to lie in the peripheral heterochromatic region of the nucleus [18–20], on average, both human alleles were observed in a central part of the nucleus with an average radial position of 0.05 to 0.25 in a scale where 1 corresponds to the nuclear envelope and this was true at all stages of B-cell maturation (Figure 1B). Of note, both the non-productive and productive alleles exhibited some level of heterogeneity in their radial positioning particularly prior to final maturation (Figure 1B).

**Figure 2:**
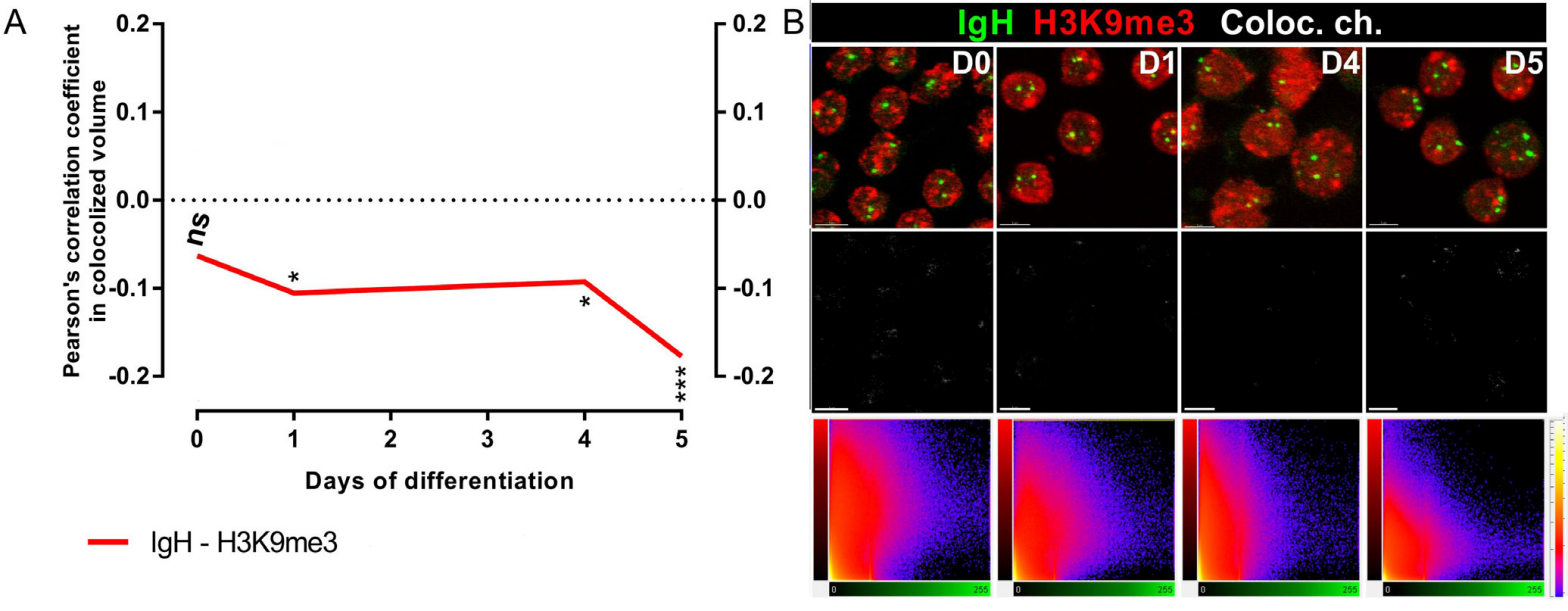
*IGH* alleles do not colocalize with heterochromatin clusters **A**. Pearson's coefficients for colocalization of *IGH* H3K9me3 and *IGH* in B-cells from Day_0_, Day_1_, Day_4_ and Day_5/6_ show the absence of colocalization. **B**. Top, Immuno-FISH with the BAC probe RP11-346I20 (green), and either DAPI (blue, B) or anti-H3K9me3 (red, C); middle, FISH signals of the IGH loci; Bottom, 2D representation of Pearson's coefficients for colocalization in Day_0_, Day_1_, Day_4_ and Day_5/6_ B-cells. Scale bar = 10 μm.

To investigate whether the human *IGH* gene loci colocalized with heterochromatin as previously reported in murine cells [18–20], we used immuno-FISH to visualize the position of the *IGH* loci relative to constitutive heterochromatin as revealed by staining with an antibody directed at histone H3 trimethylated on lysine 9 (H3K9me3). The corresponding fluorescence profiles were extracted on single confocal section images. Calculated at each stage of differentiation as described in materials and methods, the Pearson's coefficients of colocalization between *IGH* and H3K9me3 were constantly negative (Figure 2A). Representative images of immuno-FISH and the corresponding 2D histograms (fluorograms) are shown in Figure 2B. Fluorograms represent scatter plots with IGH pixel intensities plotted against intensities of the same pixels labeled with H3K9me3 (Figure 2B). Taken together, these data reveal a total lack of correlation indicating that neither of the two *IGH* alleles associated significantly with heterochromatin at any stage of human B-cell maturation.

### One *IGH* allele associates with the nucleolus and colocalizes with AID

**Figure 3:**
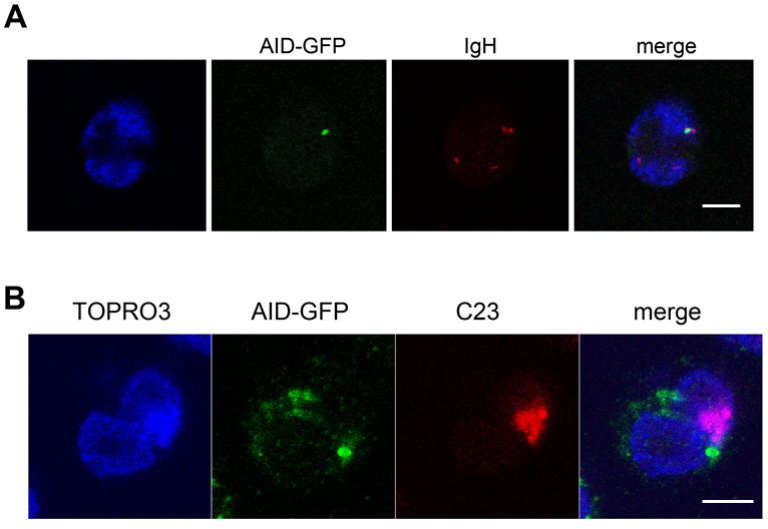
The ectopically expressed AID-GFP forms a unique perinucleolar focus associated with IGH at the periphery of nucleoli **A**., **B**., BL2 cells expressing AID-GFP fixed, hybridized and stained 48h after stimulation. Typical images of immuno-FISH staining showing the colocalization of TOPRO3, AID-GFP (green), *IGH* (red, B) or nucleolin (C23; red, C). Scale bar = 5 μm.

We have previously reported that in Mantle cell lymphoma, Burkitt lymphoma as well as in naïve B-cells, one or both *IGH* loci can be seen associated with the nucleolus [24, 25]. Here, we have hypothesized that the recombination events occurring in differentiating B-cells could take place in the perinucleolar space where AID could colocalize with the *IGH* locus under rearrangement. AID cannot be detected in normal B-cells due to its extremely low level of expression [26]. To circumvent this difficulty, we capitalized on a previously described cell line derived from the BL2 Burkitt lymphoma cell-line in which expression of an AID-GFP fusion protein can be stimulated by addition of IL-4 [27]. In the present experimental system, AID could then be detected starting at 48 hours after induction when it was visualized as a single body (Figure 3A-3B) adjacent to the nucleolus (Figure 3B) and colocalizing with one *IGH* locus (Figure 3A), thus indicating that in this *in vitro* system, an *IGH* gene locus can associate with an AID-containing body in a compartment in close proximity with the nucleolus.

### One or both *IGH* alleles associate with the nucleolus at distinct stages of human B-cell maturation

**Figure 4:**
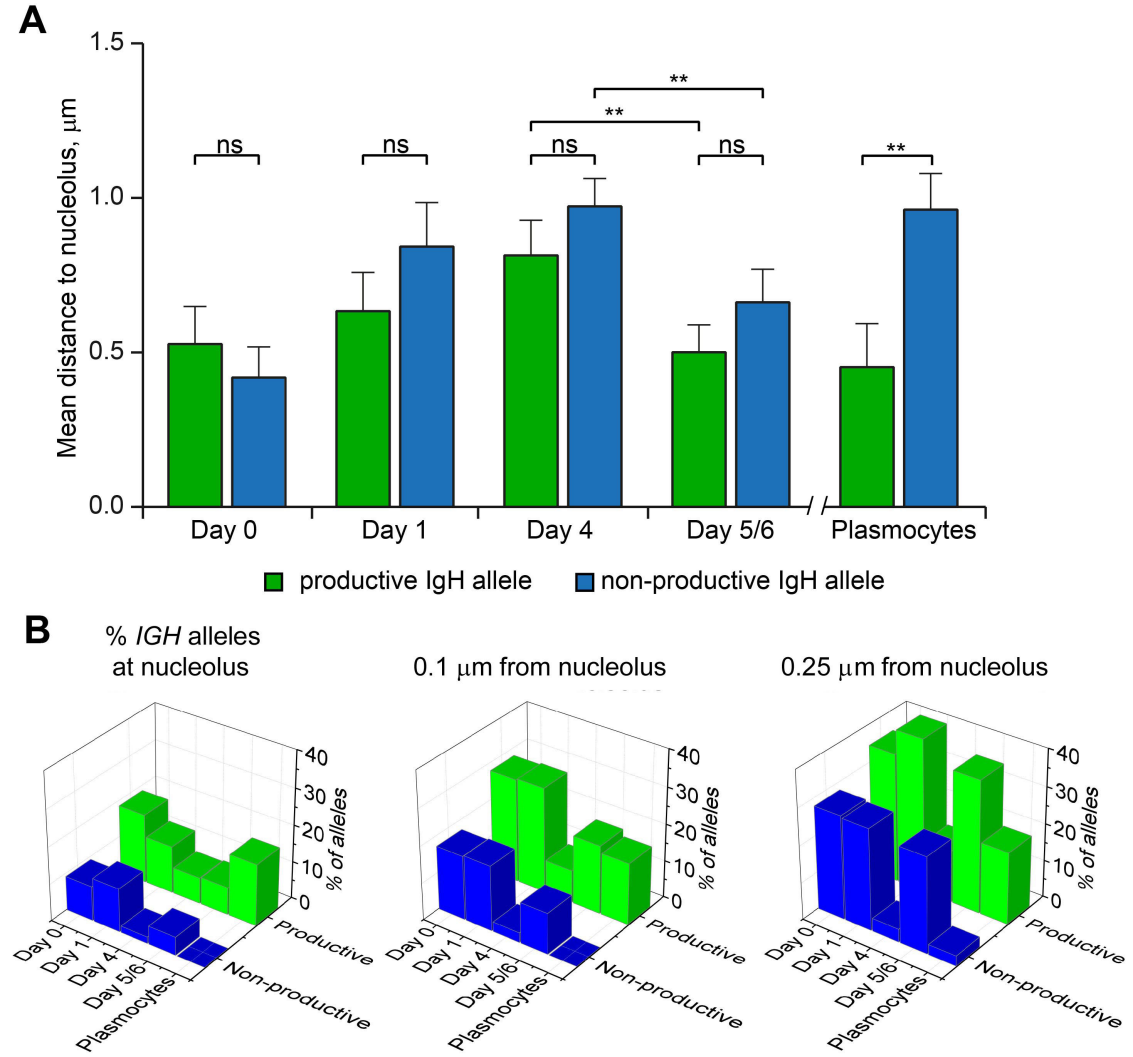
Localization of productive and non-productive *IGH*-alleles relative to nucleolus at different stages of B-cell differentiation **A**. Mean distances between nucleolus and the *IGH* alleles measured in the nuclei of Day_0_ cells (*n* = 76), Day_1_ cells (*n* = 102), Day_4_ cells (*n* = 113), Day_5/6_ cells (*n* = 81), and Plasmocytes (*n* = 37). Error bars represent 95% CI. P-values were calculated using Tukey's multiple comparison test (ns, non-significant; ** <0.01); adjusted p-values for all pairs of means are presented in Table S2. **B**. Percentage of productive and non-productive *IGH*-alleles localized at nucleolus (left), within a distance of 0.1 µm to nucleolus (middle) or within a distance of 0.25 µm to nucleolus (right), as calculated for the cells analyzed in (A).

With AID observed in the perinucleolar region, we next investigated whether the *IGH* gene locus could possibly relocalize towards the perinucleolar space at the onset of SHM and CSR. We used two-color FISH to detect the productive and non-productive *IGH* alleles and measure their proximity with the nucleolus in differentiating B-cells. Proximity was defined when the distance between the center of the *IGH* FISH signal and the outer limit of the nucleolus was less than 0.25 μm. In naïve B-cells, both *IGH* alleles were localized at a distance less than 0.5 μm from the limit of the nucleolus on average (Figure 4A). Both alleles moved progressively away from the nucleolus until Day_4_ of differentiation (Table S2 and Figure 4A). At Day_5/6_, however, the average distance of the *IGH* alleles to the nucleolus suddenly diminished as compared to Day_4_ and this was true for both the productive and non-productive alleles (p<0.01, Figure 4A). Interestingly, the situation was different in plasmocytes where a statistically significant difference (p<0.01) existed between the productive *IGH* allele which remained adjacent to the nucleolus (Figure 4A) and the non-productive allele which again was localized away from the nucleolus (Figure 4A). Next, the percentages of alleles located at the limit of the nucleolus or at a distance less than 0.1 or 0.25 μm from the nucleolus were calculated at the different stages of differentiation. As can be seen in Figure 4B, more productive than non-productive *IGH* alleles were present in each category at each stage of differentiation (compare green and blue boxes in each of the three panels). Again this was particularly striking in plasmocytes (Figure 4B). This representation also shows clearly that a general movement towards the nucleolus is triggered for both the productive and non-productive *IGH* alleles between Day_4_ and Day_5/6_ of differentiation, i.e. at the onset of SHM and CSR (see in particular Figure 4B, right panel).

### The nucleolus-associated *IGH* allele colocalizes with γH2AX foci during somatic hypermutation and class-switch recombination

**Figure 5:**
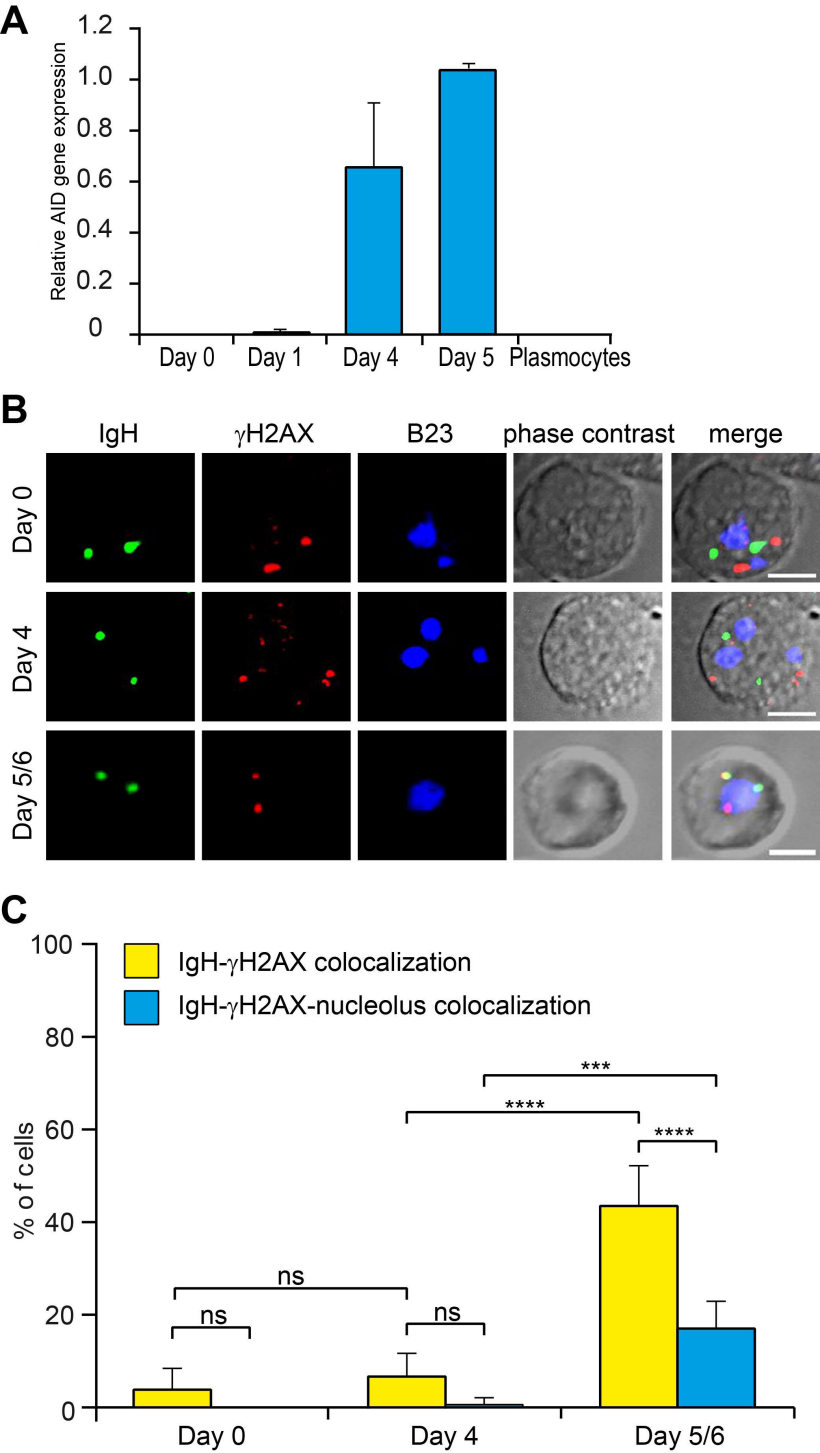
*IGH* colocalizes with the nucleolus-proximal γH2AX foci in Day_5/6_ B-cells **A**. AID expression in differentiating B-lymphocytes measured by qPCR relative to GAPDH expression. **B**. Staining of γH2AX foci in differentiating B-lymphocytes. *IGH* (green), γH2AX (red) and nucleoli (B23, blue) were simultaneously revealed by immuno-FISH. Scale bar = 5µm. **C**. Percentage of cells containing the *IGH* locus colocalizing with γH2AX or with both γH2AX and nucleolus. Columns represent means with 95% CI (error bars) for several independent calculations (Day_0_, *n* = 10; Day_4_, *n* = 8; Day_5/6_, *n* = 10). P-values were calculated using Tukey's multiple comparison test (ns, non-significant; *** <0.001; **** <0.0001).

AID is the enzyme crucial for SHM and CSR of immunoglobulin genes [3] which correspond to the final stages of immunoglobulin maturation during B-cell differentiation into plasmocytes but due to its extremely low concentration, AID cannot be visualized by immunostaining. Using RT-PCR, we found that the expression of AID started at Day_4_ and further increased at Day_5/6_ in the *in vitro* system of B-cell differentiation used here (Figure 5A). We next visualized simultaneously the *IGH* alleles, the nucleolus and DNA double strand breaks as revealed by γH2AX foci (Figure 5B). As described elsewhere, this modification of the H2AX histone provides a marker of the DNA breaks induced by AID during SHM [28]. It can be seen in Figure 5C that a γH2AX focus, presumably resulting from the action of AID colocalized with an *IGH* gene locus in up to 44% of the cells at Day_5/6_ vs. 7% at Day_4_. Among these colocalized stainings, a further colocalization with a nucleolus as revealed using the B23 antibody was observed in 19% of the cells at Day_5/6_ vs only 1% at Day_4_. On Day_5/6_ the colocalization frequency of γH2AX foci with the productive or non-productive *IGH* alleles was analyzed separately, indicating that the colocalization occurred more frequently with the productive (in ~70% of the cells) than the nonproductive (~30%) *IGH* allele (data not shown).

## DISCUSSION

Somatic hypermutation and class switch recombination depend upon the action of a highly mutagenic enzyme, AID, whose activity creates double strand breaks within immunoglobulin genes in differentiating B-cells. It is therefore not surprising that AID be expressed at a very low level which is one way to prevent off-target activities which would prove highly deleterious through mutations, translocations and possibly cell death [29, 30]. Another way to reduce the risks associated with this enzyme would be to concentrate it in precisely restricted areas of the nuclear space. Here we have tackled the hypothesis that AID-associated gene rearrangements occur in specific AID-containing compartments. The existence of such recombination compartments has been proposed as soon as the 80s for CSR [31] but it has never been experimentally demonstrated. Here, we have studied the compartmentalization of recombination using an experimental system developed by Fest and collaborators [21]. In this *ex vivo* system, events occurring in lymph nodes are recapitulated with specific stimuli activating B-cells prior to inducing SHM and CSR. We used 3D-FISH in combination with immunofluorescence staining to explore the kinetics of nuclear positioning of the two *IGH* gene loci in normal B-cells isolated from peripheral blood and induced to activate for 5 to 6 days *in vitro*. We did not observe any major difference between the productive and non-productive *IGH* alleles which exhibited a somewhat variable positioning from the center of the nuclei towards their periphery at all stages investigated until Day_4_. Despite this heterogeneity, the pattern of *IGH* localization changed at Day_5/6_ with a statistically significant movement towards the center of the nucleus for both the productive and non-productive alleles. This more central localization of the two *IGH* alleles was even more evident in plasmocytes. This lack of overt difference between the two *IGH* alleles in this human system contrasts with the murine situation where the non-productive *IGH* allele, but not its productive counterpart, has been reported to associate with peripheral heterochromatin [18–20]. Here, using a staining antibody directed to histone H3 trimethylated on lysine 9, a mark of heterochromatin, we have found no sign of such colocalization with any of the two *IGH* gene loci.

Still, our analysis of the nuclear positioning of the two *IGH* loci has clearly evidenced the variability and mobility of these genes crucial for the physiology of differentiating B cells. Interestingly, in non-lymphoid cells, the *IGH* locus forms a peripheral lamin-associated domain (LAD) [32]. Their repositioning towards the center of the nucleus observed in B-cells might merely indicate their newly acquired association with centrally positioned transcription factories [33]. It is known that genes can change their nuclear ‘neighborhoods’ by re-positioning from a repressive site to a transcriptionally active compartment, and *vice versa* during cell differentiation. Indeed, their relocation towards transcription factories occurs by extrusion of decondensing chromatin loops into interchromosomal spaces where they can intermingle with neighboring chromosome territories [34]. In the system analyzed here, transcription of the *IGH* locus is essential for both SHM and CSR as it is required for AID to function through deamination of single-stranded DNA [35]. We thus propose that the changes in the radial position of the *IGH* gene loci are associated with recombination events taking place in specific nuclear compartments.

The perinucleolar space could serve as such a recombination compartment. Indeed the enzymes involved in immunoglobulin gene rearrangements, be they at the earlier stage of V(D)J recombination in the case of the RAG1/2 enzymes or at the later AID-dependent step, have been shown to accumulate in the nucleolus [22, 23]. Moreover, the *IGH* gene locus has been reported as belonging to so-called nucleolus-associated domains (NADs) [36]. We interpret the data presented here as an indication that the *IGH* gene locus associates with the nucleolus in lymphoid cells in specific association with recombination events. The main function of the nucleolus is to transcribe rRNA for ribosome biogenesis but it is also implicated in the cellular response to stress and in the regulation of the cell cycle (reviewed in [37]. As proteins involved in *IGH* gene recombination are also deposited in the nucleolus [22, 23], it is tempting to speculate that the nucleolus plays an additional role in genetic recombination, e.g. through the release in the perinucleolar space of recombination enzymes at specific times during B-cell maturation. Here, we have found that both the productive and nonproductive *IGH* alleles were located near the nucleoli on Day_5/6_ of differentiation, the exact time when SHM and CSR take place in our experimental system. SHM within the variable segments of the immunoglobulin genes occurs during at the terminal phase of B-cell differentiation, i.e. at Day_5/6_ in our system. SHM is activated by AID which indirectly triggers the creation of double strand breaks in the *IGH* gene locus. Using an antibody detecting γH2AX foci as a marker of the AID-induced DNA breaks [28], we observed γH2AX foci at Day_4_ and even more importantly at Day_5/6_ which was the time when their localization in the vicinity of a nucleolus had become striking. Of note, even at this time point, we did not observe any significant difference in localization relative to the nucleolus between the two *IGH* alleles. A previously reported 4C-seq analysis of both the productive and non-productive alleles also indicated that allelic exclusion does not appear to be associated with the intranuclear position of the respective loci [38]. This is quite consistent with the notion that SHM is a process that applies to both the productive and non-productive *IGH* alleles [39]. Our results thus suggest the existence of a specific recombination compartment located in the perinucleolar space. Further experiments will be necessary to explore the actual relevance of this compartment and to investigate its role in SHM and CSR of immunoglobulin genes.

## MATERIALS AND METHODS

### *In vitro* B-cell differentiation

Peripheral blood cells from healthy volunteers were obtained from the French Blood Center. Naive B cells (Day_0_; CD19^+^CD27^-^) were purified from peripheral blood mononuclear cells by depletion with magnetic beads (naive B cell isolation kit II, Miltenyi Biotech). Then the cells were activated in RPMI 1640 (Invitrogen) supplemented with 10% FCS and antibiotics (Invitrogen). Purified B-cells were cultured at 7.5х10^5^ cells/ml in 24-well plates and stimulated during 4 days with 2 µg/ml F(ab’)2 Fragment Goat Anti-Human IgA+IgG+IgM (H+L) (Jackson ImmunoResearch Laboratories, West Grove, PA), 50 ng/ml recombinant human soluble CD40L associated with 5 µg/ml cross-linking Ab (R&D Systems, Abingdon, United Kingdom), 2.5 µg/ml CpG oligodeoxynucleotide 2006 (Cayla Invivogen, Toulouse, France) and 50 U/ml recombinant IL-2 (SARL Pharmaxie, Aigueperse, France). To initiate plasmablast generation, the cells were harvested on Day_4_, washed, and seeded at 4 × 10^5^/ml with IL-2 (50 U/ml), IL-4 (10 ng/ml), and IL-10 (10 ng/ml) (R&D Systems). Mature plasmocytes were isolated from peripheral blood using the CD138^+^ Plasma Cell Isolation Kit (Miltenyi Biotech).

The *in vitro* model of B cell differentiation display differentiation patterns and transcriptional profiles corresponding to the initiation of plasmocyte differentiation from cycling B precursors like it appears in human germinal centers [21]. The *in vitro* time points correspond to the steps when B cells start to proliferate while migrating to the follicle interior: on Day_1_ (22h after activation) B cells proliferate like in primary follicle and to the Day_4_ usually triple in number like in the Dark Zone of germinal center, and at the Day_5/6_ the cells display a transition of centrocytes to the Light Zone of germinal center with maximal AID expression.

### Cells

The Burkitt Lymphoma BL2 cell line expressing AID-GFP fusion protein was grown in suspension in RPMI-1640 medium, supplemented with 10% complement-inactivated fetal calf serum, L-glutamine (2mM), penicillin (100 U/ml) and streptomycin (100 mg/ml) in 5% CO_2_ in a humidified atmosphere at 37°C. The AID expression was stimulated by addition of 20 ng/ml IL-4 as described elsewhere [27] and the cells were fixed 48h after the stimulation.

### Three-dimensional fluorescence *in situ* hybridization (3D-FISH) and immuno-detection

Cells were immobilized on Cell-Tak (BD Biosciences, Bedford, MA)-coated glass coverslips. The slides were then treated as described previously to preserve their 3D structure. Denatured nuclei were hybridized overnight with denatured probes. The probes used in this study were RP11-346I20 that recognizes the constant region of the *IGH* locus and RP11-259B19 for the J/D region. The probes were purchased stained from Blue Gnome (Cambridge, UK). After probe hybridization, slides were washed according to the manufacturer's protocol. Nucleoli where detected using mouse anti-B23 and anti-C23 antibodies (Sigma, St Louis, MO) and chicken anti-mouse Alexa 647 (Molecular Probes, Carlsbad, CA). Active RNA-pol II was detected using rabbit anti-RNA pol II antibodies (Active motif) and goat anti-rabbit antibodies. Heterochromatin was detected using rabbit anti-H3K9me3 antibodies (Active motif) and goat anti-rabbit secondary antibodies.

### Confocal microscopy

Nuclei were scanned with an axial distance of 100 nm using a laser scanning confocal microscope (Zeiss LSM 510, Zeiss, Oberkochen, Germany). Stacks of gray-scale two-dimensional images were obtained with a pixel size of 47 nm. Displayed overlays of confocal images were processed with ImageJ (Rasband, W.S., ImageJ, U. S. National Institutes of Health, Bethesda, Maryland, USA, http://rsb.info.nih.gov/ij/).

### Image processing

Measurements and Statistical Analysis. The images obtained using Zeiss LSM 510 were analyzed using semi-automated image processing and analysis tools as described elsewhere [24, 25, 40, 41]. In brief, gene alleles were detected using an automatic threshold segmentation procedure with subsequent subtraction of a background image generated. Radial position of each gene allele was obtained by computing a tri-dimensional “orbit” of the corresponding object in the nucleus. The radial position of each object was obtained as proportion of the volume delimited by the “orbit” to the total nuclear volume. The value 0 corresponds to the central position of a gene in the nuclear volume. Nucleoli were detected using automatic threshold segmentation as described above. Distributions were compared using the Student's *t* test.

### RT-qPCR

RNA was extracted using RNeasy microkit (Qiagen) and reverse transcribed into cDNA with Superscript II (Invitrogen). Quantitative RT-PCR (qRT-PCR) was performed using the TaqMan Universal Master Mix and assays-on-demand from Applied Biosystems (Foster City, CA). Gene expression levels were quantified using HPRT1 as endogenous control. The 2 exp(−ΔΔCt) method was used to determine the relative expression of AID.

### Ethics statement

The human samples were obtained in accordance to the National legislation.

## SUPPLEMENTARY MATERIALS TABLES






