# Supplementary material for: The IGH locus relocalizes to a “recombination compartment” in the perinucleolar region of differentiating B-lymphocytes
Source: Oncotarget. 2017 Apr 7;8(25):40079–89. doi: 10.18632/oncotarget.16941 (PMC5522243; doi:10.18632/oncotarget.16941)
Supplement: Supplementary file 1 [file oncotarget-08-40079-s001.pdf]

The *IGH* locus relocalizes to a "recombination compartment" in the perinucleolar region of differentiating B-lymphocytes

Supplementary Material

For Supplementary Tables see in Supplementary Files
